# Development of a Community-Wide Cardiovascular Risk Reduction Assessment Tool for Small Rural Employers in Upstate New York

**Published:** 2009-03-15

**Authors:** Rivka C. Liss-Levinson, Ron Z. Goetzel, Nanette Goodman, James X. Kennedy

**Affiliations:** Institute for Health and Productivity Studies, Emory University, At the time of this study, Ms Liss-Levinson was affiliated with Cornell University’s Institute for Policy Research, Washington, DC; Rollins School of Public Health, Emory University, Atlanta, Georgia and Thomson Reuters, Washington, DC. At the time of this study, Dr Goetzel was affiliated with Cornell University’s Institute for Policy Research, Washington, DC; Washington, DC. At the time of this study, Ms Goodman was affiliated with Cornell University’s Institute for Policy Research, Washington, DC; Community Health Foundation of Central & Western New York, Syracuse, New York. At the time of this study, Mr Kennedy was Executive Director of the Cayuga Community Health Network, Inc

## Abstract

Employers are implementing workplace health promotion programs that address modifiable health risk factors such as overweight and obesity, smoking, high blood pressure, high cholesterol, physical inactivity, poor diet, and high stress. Research with large employers has found that these programs can improve workers' health and decrease the costs associated with medical care, absenteeism, and presenteeism. Despite their promise, health promotion programs are not widely embraced by small businesses, especially those in rural communities. This article reviews the barriers encountered by small and rural businesses in implementing health promotion programs. We describe an approach developed in cooperation with the New York State Department of Health's Healthy Heart Program and the Cayuga Community Health Network to engage small businesses in health promotion. We review the development and implementation of an assessment tool created to evaluate current workplace health promotion programs, policies, and practices targeting cardiovascular disease among small, rural employers in upstate New York. Potential benefits of the assessment tool are discussed, and the instrument is made available for the public.

## Introduction

Harris and colleagues recently presented the results of a pilot study in which 8 large employers from the Pacific Northwest, ranging in size from 7,500 to 115,522 employees, were recruited into a marketing study aimed at increasing adoption of evidence-based practices focused on cancer prevention (1). The researchers were successful in persuading employers to adopt several of these practices but noted that work is needed to engage the approximately 200,000 small businesses (those with fewer than 100 employees) that employ about half of the US workforce in similar efforts. Although their research focused on adopting evidence-based policies, programs, and practices directed at cancer prevention, many of the same issues apply to the prevention of cardiovascular disease (CVD).

Evidence is mounting that individualized counseling provided by large employers that is focused on CVD prevention can achieve significant and sustained reductions in risk factors for heart disease and stroke (2-3). Effective individual interventions include medical screenings, health risk assessments (HRAs) and referrals (4), individual follow-up, education, self-management, skill building, and coaching and counseling (5). These individualized interventions can be buttressed by environmental and ecological changes introduced at the workplace. Such changes include large-scale social marketing initiatives (eg, posters, memos, e-mails) that are consistent, are frequent, and have a simple message (4-6); health education classes, workshops, and support groups with individual goal setting (7); financial and other incentives that encourage employees to participate in prevention activities (8); and corporate policies that support a healthy lifestyle and achievement of health behavior goals (9). These multicomponent, well-resourced, and well-implemented programs have been shown to improve employee health, achieve cost savings, and heighten productivity among workers at large companies (2,10).

Several best and promising practices have been identified in the health promotion literature. For example, a more comprehensive approach to worksite health promotion across multiple risk factors is preferred to one that adopts a single focus, targeting a subset of the employee population with a particular risk (2,11). Other promising practices include achieving organizational commitment, offering incentives to participate, providing effective screening and triage, having state-of-the-art interventions, and providing ongoing program evaluation (12). Several sources have identified other key organizational factors associated with successful outcomes (13-15).

Recommendations from 2 government-supported task forces (16-17) can help guide employer efforts in designing and implementing evidence-based prevention and health promotion programs. The first, the US Preventive Services Task Force (USPSTF), sponsored by the Agency for Healthcare Research and Quality, reviews the literature and makes recommendations regarding the effectiveness and applicability of clinical preventive services that employers should incorporate into their medical benefit plan designs. The second, the Centers for Disease Control and Prevention (CDC) Task Force on Community Preventive Services (*The Community Guide*) (18), also relies on extensive literature reviews when making recommendations regarding the effectiveness and applicability of policies, procedures, and programs that promote health and reduce disease at the community level; it considers the workplace to be an important setting for community-based interventions.

However, very little research has been directed at the application of evidence-based programs and practices to small businesses so that they too can achieve outcomes comparable to those attained by larger employers. This may be because small employers are often limited in the expertise and experience necessary to design, implement, and evaluate evidence-based programs on their own.

We report on an initiative begun by the Institute for Health and Productivity Studies at Cornell University in association with the New York State Department of Health (NYS DOH) and the Cayuga Community Health Network (CCHN) to engage small businesses in rural communities that wish to adopt evidence-based chronic disease prevention practices. We first describe some of the challenges faced by small, rural employers and the approach taken by the NYS DOH to raise awareness of the need for workplace health promotion programs with the ultimate aim of engaging them in activities to reduce CVD among their employees.

## Characteristics of Small Employers in the United States

Small firms, defined as those with fewer than 500 workers, make up about 99% of employers in America and employ more than half of the private sector workforce (18). In New York State, there are more than 500,000 worksites with about 7.5 million workers. Of those, about 450,000 worksites (89%) have fewer than 500 employees and 63% of employees work in companies with fewer than 500 employees. Many of these smaller employers lack the resources to implement effective worksite health promotion programs.  

According to the 2004 National Worksite Health Promotion survey, only 11% of worksites with 50 to 99 employees used HRAs compared with 45% of those with more than 750 employees (19). The same survey found that fewer than 5% of sites with 50 to 99 employees, compared with 24% of sites with more than 750 employees, offered "comprehensive" workplace health promotion programs that included health education, links to related employee services, supportive physical and social environments for health improvement, integration of health promotion into organizational culture, and employee screenings with adequate treatment and follow-up (19).

A 2005 survey of more than 2,000 firms identified a sharp divide between small employers (3-199 workers) and larger firms (5,000 or more). Almost half of large companies provided on-site fitness or health facilities, compared with 8% of small firms. Similarly, larger firms were more likely than small firms to offer smoking cessation programs, injury prevention programs, and weight-loss programs (20). Another survey of small employers in southern California found that the smallest firms (fewer than 100 employees) had fewer health promotion programs, which were most commonly safety-related and mandated by law (21).

## Challenges Facing Small Employers

The disparity between large and small employers is not surprising since small businesses confront significant challenges in implementing health promotion programs. The lack of dedicated resources is a key obstacle for small businesses. Most have limited human resources staff. Thus, they are unlikely to have experts who know how to design and organize a health promotion program or who can commit the time to develop those skills (21). This is problematic since worksites with a staff person assigned to health promotion are 10 times more likely than sites without a dedicated resource to have a comprehensive health promotion program, regardless of worksite size (19). Further, small businesses tend to have lower profit margins than larger employers, making it difficult for them to invest in and sustain health promotion programs (22). Often, health and safety regulations overwhelm small businesses, making them unlikely to establish health-related programs not mandated by law (21).

Small employers also have limited access to commercial worksite vendors. Although some health insurance providers offer some forms of health promotion programs, these are often limited and only provided through the medical care system. Furthermore, many small businesses do not offer health insurance. While 99% of large firms (200 or more workers) offered health benefits in 2007, only 59% of small firms (3 to 199 workers) did (23). Small employers that do offer insurance often purchase their fully insured policies from a third-party broker or small insurer, whereas large employers are more likely to be self-insured and thus more engaged in managing their employees' health and health care utilization. Almost half of businesses with more than 100 employees are self-insured compared with 17% of companies with fewer than 100 employees (24). Often these small employers have limited power to negotiate with health insurers on which services the plan covers.

Small businesses in rural settings face a unique set of challenges to promoting healthier lifestyles. Representing about 20% of the US population, they are diverse in their demographic composition, availability of community resources, and public health presence. Compared with metropolitan areas, rural areas have higher rates of smoking, more high-fat diets, and higher percentages of people with sedentary lifestyles and decreased perception of heart disease risk (25). Furthermore, companies in rural settings have limited access to clinical preventive services. Although 20% of the US population lives in rural areas, only 10% of all physicians practice in those areas, often leading to a shortage of specialists who can provide certain clinical preventive services, such as mammograms and colonoscopies. As a result, rural residents may need to travel long distances to reach the nearest service provider (26).

## Health Promotion Opportunities for Small, Rural Employers

Despite these challenges, small, rural employers also have certain strengths that can be leveraged to develop and sustain health promotion programs. For example, they tend to have visible and accessible senior leaders who may exemplify good health practices and become internal champions for health-promoting behaviors. In a small business, it is often easier to communicate with employees and create a sense of community among workers. Furthermore, human resources managers often develop close, trusting relationships with workers and are therefore more likely to engage them in programs that workers may otherwise find suspicious (27). Finally, when small firms implement health promotion programs, they tend to achieve better participation rates than larger firms because their programs are embedded within the company's culture and norms (21).

## Purpose

The NYS DOH, in partnership with CCHN, sought to heighten awareness among small, rural employers in upstate New York so that they would adopt evidence-based practices to prevent CVD among their employees. A first step in that process involved designing and distributing a Community-Wide Cardiovascular Risk Reduction Assessment instrument to participating organizations. The assessment asked employers to provide information about their adoption of evidence-based prevention and health promotion programs and policies, and was meant to educate them about steps they could take to achieve CVD prevention among their workers. The project, although small in scale, was meant to serve as a model for other small businesses and business coalitions that may wish to partner with their local public health agencies to design and implement workplace programs. We describe the assessment and offer it for download at www.sph.emory.edu/ihps so that other small employers in rural communities might apply the tool to their prevention efforts.

## Approach

### Develop the business case

We met with officials from the NYS DOH and CCHN to develop, in broad strokes, the elements of the assessment. We asked for and received support for recruiting small employers from CCHN. We developed a rationale and business case presentation for the CCHN, highlighting evidence for the efficacy of CVD health promotion and disease prevention programs at the workplace. The business case presentation (also available online at www.sph.emory.edu/ihps) sought to recruit employers to the initiative by providing an overview of the project, its objectives, a timetable, sample assessment questions, report templates, time commitments, and a summary of what employers would gain from the project.

CCHN began recruiting potential employers for the project by mailing invitations to more than 500 small to medium-sized businesses. In response to these efforts, 25 organizations attended the project recruitment meeting in Auburn, New York.

### Design data collection instrument

We next developed the assessment instrument, an electronic data collection tool designed to be completed in less than 2 hours. A main aim of the assessment was to draw attention to the myriad programs and policies, many of which are low or no cost, that employers can adopt to promote health in the workplace. To mitigate concerns about privacy or confidentiality, participants were assured that all information collected in the survey would remain confidential at the employer level and that results would only be provided in aggregate. To ensure that assessment questions were understandable and appropriate for the study population, 3 area employers reviewed the form and provided feedback on the overall approach and its applicability to small employers. The assessment was divided into 3 modules. For all modules, the goal was to capture key data elements that could be synthesized into clear, actionable information for organizations wishing to initiate or optimize their CVD prevention efforts.

*Module 1: Human Resource Metrics* included basic information about the type of business and characteristics of the employees. To quantify the costs that could be reduced by promoting health in the workplace, the module asked respondents to identify their expenditures associated with health care, absenteeism, short- and long-term disability, and workers' compensation.

*Module 2: Workplace Policies, Practices, and Environment* assessed current employer health promotion and disease prevention programs and policies. The main source of items used in this section was *A Purchaser's Guide to Clinical Preventive Services* (28), produced by the National Business Group on Health, which provides evidence-based recommendations to guide employers in designing their health benefit packages. The assessment asked which *Purchaser's Guide* recommended preventive services were covered by the organization's health plan. Respondents were also asked about the extent to which they instituted best practices and policy recommendations from the USPSTF and the CDC *Community Guide*. The assessment focused on policies and practices affecting CVD, including general employee health and wellness (eg, employee health promotion and disease management programs, clinical preventive services); diet and nutrition (eg, vending machines, cafeteria, and other food services; overweight and obesity); exercise and physical activity (eg, presence of fitness facilities); tobacco use (eg, smoking cessation programs and services); and stress and emotional health (eg, employee assistance programs).

*Module 3: Organizational Support* assessed the extent to which the organizations supported workplace health promotion and disease prevention efforts through senior managers' commitment to a "healthy company" culture. Employers also completed an 18-item instrument entitled "Leading by Example" or LBE (29). The LBE asked employers to note the extent to which they agreed with such statements as "our organization . . . is committed to health promotion as an important investment in human capital; . . . provides adequate financial support for health promotion; . . . shares information with employees about the effect of employee health on overall business success."

### Analyze and report

We then aggregated the data for all participating organizations and prepared a summary report for all employers. Each participating employer received a feedback report comparing the employer's data with those of the aggregate group. At the conclusion of the project, we presented our findings to the participating employers. We discussed how employers could apply their assessment findings to initiate or improve their workplace health promotion programs and policies.

## Discussion

A small pilot project initiated by the NYS DOH, a public health agency, and CCHN, a local rural health network, sought to raise awareness among employers in upstate New York about evidence-based health promotion programs and practices. The approach taken was to design and administer an assessment tool that highlighted programs and practices and ask employers whether these were in place at their organization. We expected that educating employers about these practices would prompt them to adopt more of them in their organizations. Because funding for this pilot study was limited, no follow-up was provided to determine whether the recommended practices were actually adopted.

Below, we review ways that employers can introduce evidence-based clinical and community-oriented preventive services. In terms of benefit plan design, employers can consider removing or reducing financial barriers to accessing USPSTF recommended preventive services. Furthermore, they can encourage their health plans to provide financial incentives to providers who appropriately screen patients for health risk factors such as high blood pressure, obesity, and tobacco use at each clinical encounter and for depression when indicated. Employers can monitor these activities by requesting reports from their health plan on physicians' screening rates for the risk factors.

To encourage adoption of community-based health promotion programs, employers can support programs aimed at increasing physical activity by 1) providing incentives to participate in such programs; 2) establishing a network of available, on-site physical activity group facilitators; and 3) working with community-based organizations that provide and promote physical activity, such as the American Diabetes Association Tour for the Cure, the American Cancer Society Relay for Life, or the American Heart Association Heart Walk.

Employers can offer healthy food options at company-sponsored meetings and functions; promote healthful food options; develop model healthy food and beverage vending contracts; negotiate corporate rates for worksite-based weight control programs; and develop working relationships or partnerships with local greenmarkets and farmers' markets to offer coupons and promotions.

To reduce tobacco use among workers, employers can establish partnerships with local tobacco control coalitions; establish policies for smoke-free worksites; prohibit smoking in company-owned vehicles and workplace entrances; ban sales of tobacco products on site (eg, no cigarette vending machines); and provide coverage for the treatment of nicotine addiction through the use of medications and counseling for smoking cessation.

Other actions employers can institute include issuing regular messages from senior managers about policies supporting healthy behaviors and health promotion; providing managers with training on the importance of employee health promotion; establishing performance objectives for managers related to workplace health improvement; offering free or low-cost educational materials (eg, self-care books) supporting healthy behaviors; and granting prizes, awards, and recognition for employees or managers who participate or promote participation in health promotion programs.

These actions are relatively low-cost and low-effort and can be adopted by many small employers on their own, working as part of community coalitions, or in partnership with their health plans. These initiatives may produce health improvement outcomes similar to those realized by many large employers. They also have the potential to achieve cost savings in the form of reduced health care spending, lower absenteeism rates, fewer safety incidents, and heightened worker productivity.

## References

[1] Harris JR, Cross J, Hannon PA, Mahoney E, Ross-Viles S (2008). Employer adoption of evidence-based chronic disease prevention practices: a pilot study. Prev Chronic Dis.

[2] Heaney CA, Goetzel RZ (1997). A review of health-related outcomes of multi-component worksite health promotion programs. Am J Health Promot.

[3] Pelletier K (1997). Clinical and cost outcomes of multifactorial, cardiovascular risk management interventions in worksites: a comprehensive review and analysis. J Occup Environ Med.

[4] Erfurt JC, Foote A, Heirich MA (1991). Worksite wellness programs: incremental comparison of screening and referral alone, health education, follow-up counseling, and plant organization. Am J Health Promot.

[5] Erfurt JC, Holtyn K (1991). Health promotion in small business: what works and what doesn't work. J Occup Med.

[6] Goetzel RZ, Jacobson BH, Aldana SG, Vardell K, Yee L (1998). Health care costs of worksite health promotion participants and non-participants. J Occup Environ Med.

[7] Masur-Levy P, Tavris DR, Elsey-Pica L (1990). Cardiovascular risk changes in a work-site health promotion program. J Am Diet Assoc.

[8] Harvey MR, Whitmer RW, Hilyer JC, Brown KC (1993). The impact of a comprehensive medical benefit cost management program for the city of Birmingham: results at 5 years. Am J Health Promot.

[9] Fielding JE, Mason T, Kinght K, Klesges R, Pelletier KR (1995). A randomized trial of the IMPACT worksite cholesterol reduction program. Am J Prev Med.

[10] Naydeck BL, Pearson JA, Ozminkowski RJ, Day BT, Goetzel RZ (2008). The impact of the highmark employee wellness programs on 4-year healthcare costs. J Occup Environ Med.

[11] Pelletier KR (2005). A review and analysis of the clinical and cost-effectiveness studies of comprehensive health promotion and disease management programs at the worksite: update VI 2000-2004. J Occup Environ Med.

[12] Goetzel RZ, Shechter D, Ozminkowski RJ, Marmet PF, Tabrizi MJ, Roemer EC (2007). Promising practices in employer health and productivity management efforts: findings from a benchmarking study. J Occup Environ Med.

[13] Goetzel RZ (1997). Essential building blocks for successful worksite health promotion programs. Manag Employ Health Bene.

[14] O'Donnell MP, Bishop CA, Kaplan KL (1997). Benchmarking best practices in workplace health promotion. Art of Health Promotion.

[15] Wellness Councils of America. Seven benchmarks of success.

[16] (2006). Guide to Clinical Preventive Services.

[17] Centers for Disease Control and Prevention's Task Force on Community Preventive Services (2007). The Community Guide.

[18] US Small Business Administration (2007). Frequently Asked Questions — Advocacy: The Voice of Small Business in Government.

[19] Linnan L, Bowling M, Childress J, Lindsay G, Blakey C, Pronk S (2008). Results of the 2004 National Worksite Health Promotion Survey. Am J Public Health.

[20] Gabel J, Claxton G, Gil I, Pickreign J, Whitmore H, Finder B (2005). Health benefits in 2005: premium increases slow down, coverage continues to erode. Health Aff (Millwood).

[21] McMahan S, Wells M, Stokols D, Phillips K, Clitheroe HC (2001). Assessing health promotion programming in small businesses. Am J Health Stud.

[22] Stokols D, McMahan S, Phillips K, O'Donnell M (2001). Workplace health promotion in small businesses. Health promotion in the workplace.

[23] (2007). Kaiser Family Foundation Health Research and Educational Trust. Employer Health Benefits, 2007 Annual Survey.

[24] Conlon F (2007). Medical Plan Type, Fee Arrangement, and Financial Intermediaries 2006. Compensation and Working Conditions On-line.

[25] Zuniga MD, Anderson D, Alexander K, Gamm LD, Hutchinson LL, Dabney BJ (2003). Heart disease and stroke in rural America. Rural healthy people 2010: a companion document to healthy people 2010; volume 1.

[26] Infante A, Meit M (2007). Delivering the US Preventive Services Task Force Recommendations in a Rural Health Plan (Vol. Research Brief Series W, No. 8).

[27] European Network for Workplace Health Promotion Cardiff Memorandum on workplace health promotion in small and medium sized enterprises.

[28] Campbell KP, Lanza A, Dixon R, Chattopadhyay S, Molinari N, Finch RA (2006). A purchaser's guide to clinical preventive services: moving science into coverage.

[29] Della LJ, DeJoy DM, Goetzel RZ, Ozminkowski RJ, Wilson MG (2008). Assessing management support for worksite health promotion: psychometric analysis of the leading by example (LBE) instrument. Am J Health Promot.

